# An unhealthy lifestyle and incident activity-limiting neck and back problems in university students: the Sustainable UNiversity Life (SUN) study

**DOI:** 10.1186/s12889-025-25478-y

**Published:** 2025-11-06

**Authors:** Clara Onell, Martin Asker, Helene Wiberg, Pierre Côté, Fred Johansson, Tobias Sundberg, Klara Edlund, Eva Skillgate

**Affiliations:** 1 Department of Health Promoting Science, Musculoskeletal & Sports Injury Epidemiology Center, Sophiahemmet University, Box 5605, Stockholm, 114 86 Sweden; 2 Unit for Intervention and Implementation Research in Worker Health, Institute of Environmental Medicine, Karolinska Institutet, Stockholm, Sweden; 3 Institute for Disability and Rehabilitation Research and Faculty of Health Sciences, Ontario Tech University, Oshawa, ON Canada; 4 Centre for Psychiatry Research, Department of Clinical Neuroscience, Karolinska Institutet & Stockholm Health Care Services, Region Stockholm, Stockholm, Sweden; 5 Division of Psychology, Department of Clinical Neuroscience, Karolinska Institutet, Stockholm, Sweden

**Keywords:** Students, Neck pain, Back pain, Physical inactivity, High sitting time, Meal skipping, Risky alcohol use, Risky tobacco use, Risky illicit drug use

## Abstract

**Background:**

Neck and back pain are common musculoskeletal conditions in university students and associated with unhealthy lifestyle behaviors such as physical inactivity, unhealthy dietary habits, smoking and risky alcohol use. Cohort studies have investigated the effect of a healthy lifestyle including several lifestyle behaviors on the risk and prognosis for neck and back pain. Studies of an association between an unhealthy lifestyle and musculoskeletal conditions in university students are lacking. This study aimed to assess the association between an unhealthy lifestyle and incident activity-limiting neck/back problems (ALNBP) in university students.

**Methods:**

Participants enrolled in the Sustainable UNiversity Life (SUN) study who did not report baseline ALNBP in the past three months were included (*n* = 3492). The baseline web-survey assessed unhealthy lifestyle behaviors (physical inactivity, high sitting time, meal skipping and risky use of alcohol, tobacco, and illicit drugs) with valid instruments and single-item questions. Participants with ≥ 3 unhealthy lifestyle behaviors were categorized as exposed to an unhealthy lifestyle. Responses to web-based follow-up surveys every third month until first reporting ALNBP or to the end of the one-year follow-up were used. The outcome ALNBP was defined as reporting limitations in daily activities due to a neck, low back and/or midback problem the past three months, assessed with a modified version of the Nordic Musculoskeletal Questionnaire. Crude and adjusted Cox regression models were built to assess the association between an unhealthy lifestyle and incident ALNBP, reported as a hazard rate ratio (HRR) with a 95% confidence interval (CI).

**Results:**

Sixty percent of the participants were women, and the mean age was 24.5 ± 6.0 years. A total of 574 participants (16%) were exposed to an unhealthy lifestyle at baseline. Having an unhealthy lifestyle generated an adjusted HRR of 1.35 (95% CI 1.12, 1.63) for incident ALNBP, compared to not having an unhealthy lifestyle.

**Conclusions:**

An unhealthy lifestyle, categorized as ≥ 3 unhealthy lifestyle behaviors, is associated with incident ALNBP in university students.

**Supplementary Information:**

The online version contains supplementary material available at 10.1186/s12889-025-25478-y.

## Background

Neck and back pain (NBP) are the most common musculoskeletal conditions and leading contributors to global ill-health, disability and need of rehabilitation worldwide [1–4]. The prevalence of NBP increases with age, yet a large burden is imposed also in younger populations [5], including university students [6, 7]. The prevalence of musculoskeletal pain in university students is suggested to have increased since the beginning of this century [8], and chronic pain has been estimated to affect 54% of university students [6], where NBP is most common [6, 9]. Not all musculoskeletal conditions interfere with daily functioning, but many can become activity-limiting and have a significant impact on overall health [4]. For example, musculoskeletal conditions in university students are associated with impaired academic functioning, poor sleep quality and poor health-related quality of life [10, 11].

In university students, NBP is more common among women [12], older students and students in health care programs [13]. Cross-sectional studies suggest that NBP in university students is associated with sedentary behavior [14], physical inactivity and coffee intake [15], use of digital devices [12, 16–21], anxiety, stress, and sleep disturbances [13, 14, 22, 23]. Cohort studies have identified discrimination and a poor study environment [24], previous NBP, poor back support, muscle tightness [25], sedentary behavior, computer, and mobile use [26] as potential risk factors for NBP in university students.

University students are prone to unhealthy lifestyle behaviors, including sedentary behavior, physical inactivity, and low intake of fruits and vegetables [27–30]. Such behaviors are part of a broader public health concern, as physical inactivity, unhealthy diet, tobacco use, and risky alcohol consumption are recognized contributors to chronic noncommunicable diseases and account for a substantial portion of the disease burden in Sweden [31, 32]. Moreover, individual lifestyle behaviors often cluster [33–36], and clustering of healthy or unhealthy behaviors is common among university students [34, 36], with an unhealthy lifestyle being associated with impaired quality of life [34].

Studies of non-student populations have investigated the importance of lifestyle behaviors for the risk and prognosis of NBP [37–41], where a healthy lifestyle consistently has been shown to be protective against NBP and associated with favorable prognosis among those affected. To the best of our knowledge, no studies have investigated the role of an unhealthy lifestyle for the risk of NBP in university students. Deepening the knowledge about modifiable risk factors for NBP in university students, and especially for activity-limiting conditions, is key for implementation of future preventive strategies. The aim of this study was, therefore, to investigate the association between an unhealthy lifestyle and incident activity-limiting neck/back problems (ALNBP) in university students.

## Methods

This study is based on the Sustainable UNiversity Life (SUN) study with the primary aim to identify risk and prognostic factors for mental health problems and musculoskeletal problems in university students (ClinicalTrials.gov ID NCT04465435 registration date 2020-06−17). The SUN-study is described in detail in the published study protocol [42]. Students enrolled at educational programs at eight Swedish universities in Stockholm and Örebro were invited to participate in the SUN-study. Participant recruitment was ongoing between August 2019 and December 2020. Participants were recruited at university campuses in 2019, where study staff gave classroom presentations explaining the study’s purpose and significance. Following the outbreak of the COVID-19 pandemic in 2020, physical recruitment was replaced with digital methods, including online presentations or email invitations. All eligible students received an invitation through email with a link to a baseline web-survey. Included participants received four follow-up surveys with three-month intervals over one year. To ensure accessibility, the surveys were provided both in Swedish and English. Data collection finished in December 2021, when the last group of students responded to the fourth follow-up survey. The SUN-study was conducted according to the Helsinki Declaration, obtained ethical approval by the Swedish Ethical Review Authority (reference number 2019–03276 and 2020 − 01449) and all participants provided informed consent electronically prior to enrollment in the study.

### Study population

**Fig. 1:**
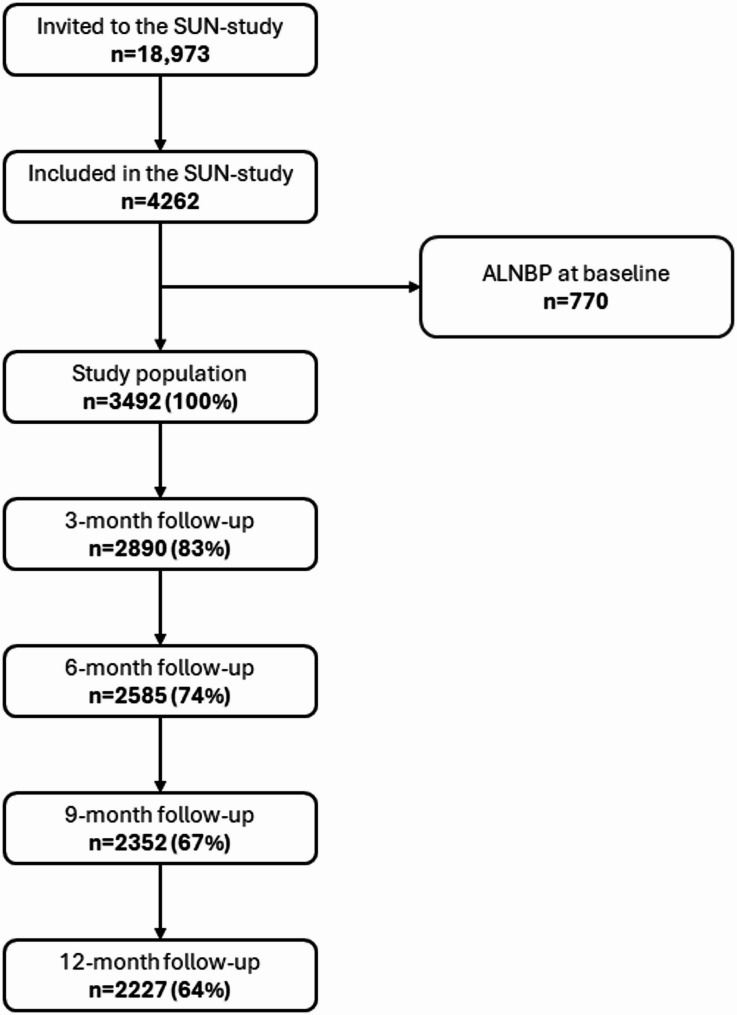
Flowchart of participant inclusion and follow-ups. Abbreviations: ALNBP, activity-limiting neck/back problems; SUN-study, Sustainable UNiversity Life study

A total of 18,973 university students were invited to the SUN-study, and 4262 students (22%) agreed to participate. At baseline, 770 participants reported ALNBP the past three months and were therefore excluded from the analyses, resulting in a cohort of 3492 participants. Participants were included until the first follow-up with an event of ALNBP, loss to follow-up or end of the one-year study period, whichever came first. Inclusion and follow-up in this study is presented in Fig. 1.

### Baseline measures

#### Physical activity

Two questions from the National Board of Health and Welfare [32] in Sweden were used to assess physical activity. Weekly minutes of exercise (e.g. running, ball sports) and daily activities (e.g. walking, cycling) during a normal week were reported. Total physical activity was calculated as a sum of minutes per week in exercise and minutes per week in daily activities, with exercise minutes doubled to account for intensity. Physical inactivity was categorized as physical activity < 150 min per week [43].

#### Sitting time

Sitting time was assessed with the SED-GIH single question [44], which has shown excellent reliability but poor validity, in line with other common questionnaires assessing sitting time. Habitual daily sitting time, excluding sleep, was reported in hours. High sitting time was categorized as sitting ≥ 10 h per day [44].

#### Meal frequency

Meal frequency was assessed with questions about habitual intake frequency of main meals breakfast, lunch, and dinner, reported as 0–7 days per week. Meal skipping was categorized as skipping at least one of the main meals ≥ 4 days per week [45].

#### Alcohol, tobacco, and illicit drug use

Alcohol, tobacco and illicit (non-medical) drug use were assessed with the Alcohol, Smoking and Substance Involvement Screening Test (ASSIST), which has shown acceptable validity for identifying varying degrees of use [46, 47]. The ASSIST generates a score of 0–42 categorized as low, moderate, or high-risk of problems or dependence. Risky alcohol, tobacco, and illicit drug use, respectively, were categorized as a moderate or high-risk score for alcohol, tobacco, and illicit drugs.

### Exposure: unhealthy lifestyle

The separate unhealthy lifestyle behaviors physical inactivity, high sitting time, meal skipping, and risky use of alcohol, tobacco, and illicit drugs, respectively, were combined into a total of 0–6 unhealthy lifestyle behaviors, with ≥ 3 unhealthy lifestyle behaviors (the highest quantile) categorized as exposure to an unhealthy lifestyle.

### Outcome: activity-limiting neck/back problems

The outcome ALNBP was evaluated at each follow-up with a modified version of the Nordic Musculoskeletal Questionnaire (NMQ) as being limited in normal daily activities (e.g. studies, hobbies) in the past three months due to trouble (ache, pain, discomfort) in the neck, low back and/or midback [48]. The questionnaire was modified to allow measurements every third month for one year and asked about the past three months instead of the past twelve months as in the original NMQ. The NMQ has been widely used in the Nordic countries, and reliability tests with test-retest have been conducted with adequate results (0–23% of non-identical answers) as well as validity tests against clinical history (0–20% of non-identical answers) [48].

### Statistical analyses

The association between an unhealthy lifestyle and the first event of ALNBP was estimated using crude and adjusted Cox proportional hazard regression models and presented as a hazard rate ratio (HRR) with a 95% confidence interval (CI). Plots of the Schoenfeld and Martingale residuals indicated proportional hazards and no nonlinearity or influential observations. Months since baseline was the underlying timescale. A priori, age (continuous), gender (woman or other), and highest parental education (at least one parent with a university degree, proxy for socioeconomic status) assessed at baseline were included covariates in the analysis. These, and other factors considered but not included in the final analysis, are presented in a directed acyclic graph (DAG) in Supplemental Fig. 1. To visualize the proportion of exposed and unexposed participants with the outcome at each follow-up, Kaplan–Meier survival curves were generated based on the adjusted Cox proportional hazard regression model. The curves were estimated using the conditional method, which estimates survival probabilities for a hypothetical individual with fixed baseline covariate values selected to represent a typical participant profile in the sample. As a sensitivity analysis, an E-value was calculated to estimate the magnitude of a confounder association that could produce bias equal to the observed association, i.e. the strength of association that an unmeasured confounder would need to have with both the exposure and the outcome, conditional on the covariates, to explain away the association [49]. Data management and analyses were performed in R version 4.2.1 (2022-06−23 UCRT).

## Results

Descriptive characteristics of the included participants are presented by exposure status in Table 1. Most participants were young women, enrolled in a medicine/health or technology education program, born in Sweden and had at least one parent with a university degree. Also, the vast majority did not report moderate or severe symptom levels of mental health problems (depression, anxiety, and stress).

**Table 1:** Baseline participant characteristics presented by exposure status

	Exposed to an unhealthy lifestyle^1^ (*n* = 574)	Unexposed to an unhealthy lifestyle (*n* = 2918)
Age, mean years ± SD	24.0 ± 5.3	24.6 ± 6.1
Gender, *n* (%)		
Woman	328 (57)	1760 (60)
Man	241 (42)	1143 (39)
Other	5 (1)	15 (< 1)
Type of education, *n* (%)		
Medicine/health	218 (38)	1335 (46)
Technology	283 (49)	1221 (42)
Social science	50 (9)	230 (8)
Economy	20 (3)	82 (3)
Other	3 (1)	50 (2)
Born outside Sweden, *n* (%)	133 (23)	613 (21)
Living alone, n (%)	179 (32)	793 (27)
≥One parent with university degree, *n* (%)	411 (72)	2142 (73)
≥Moderate depression symptoms^2^, *n* (%)	232 (40)	659 (23)
≥Moderate anxiety symptoms^2^, *n* (%)	136 (24)	422 (14)
≥Moderate stress symptoms^2^, *n* (%)	157 (27)	525 (18)
Physical inactivity^3^, *n* (%)	355 (62)	431 (15)
High sitting time^4^, *n* (%)	435 (76)	1006 (34)
Meal skipping^5^, *n* (%)	445 (76)	713 (24)
Risky alcohol use^6^, *n* (%)	213 (37)	209 (7)
Risky tobacco use^6^, *n* (%)	345 (60)	446 (15)
Risky illicit drug use^6^, *n* (%)	112 (20)	58 (2)

^1^Having ≥ 3 unhealthy lifestyle behaviors (i.e. physical inactivity, high sitting time, meal skipping, risky alcohol use, risky tobacco use and risky illicit drug use)

^2^Depression, Anxiety and Stress Scale 21 [50]

^3^Total physical activity < 150 min per week

^4^Daily sitting ≥ 10 h per day

^5^Skipping breakfast, lunch and/or dinner ≥ 4 days per week

^6^Alcohol, Smoking and Substance Involvement Screening Test [46]

At baseline, a total of 574 participants (16%) were exposed to an unhealthy lifestyle. Of these, 355 (62%) were categorized as physically inactive, 435 (76%) had a high sitting time, 445 (76%) skipped meals, 213 (37%) had a risky alcohol use, 345 (60%) had a risky tobacco use and 112 (20%) had a risky illicit drug use.

**Fig. 2:**
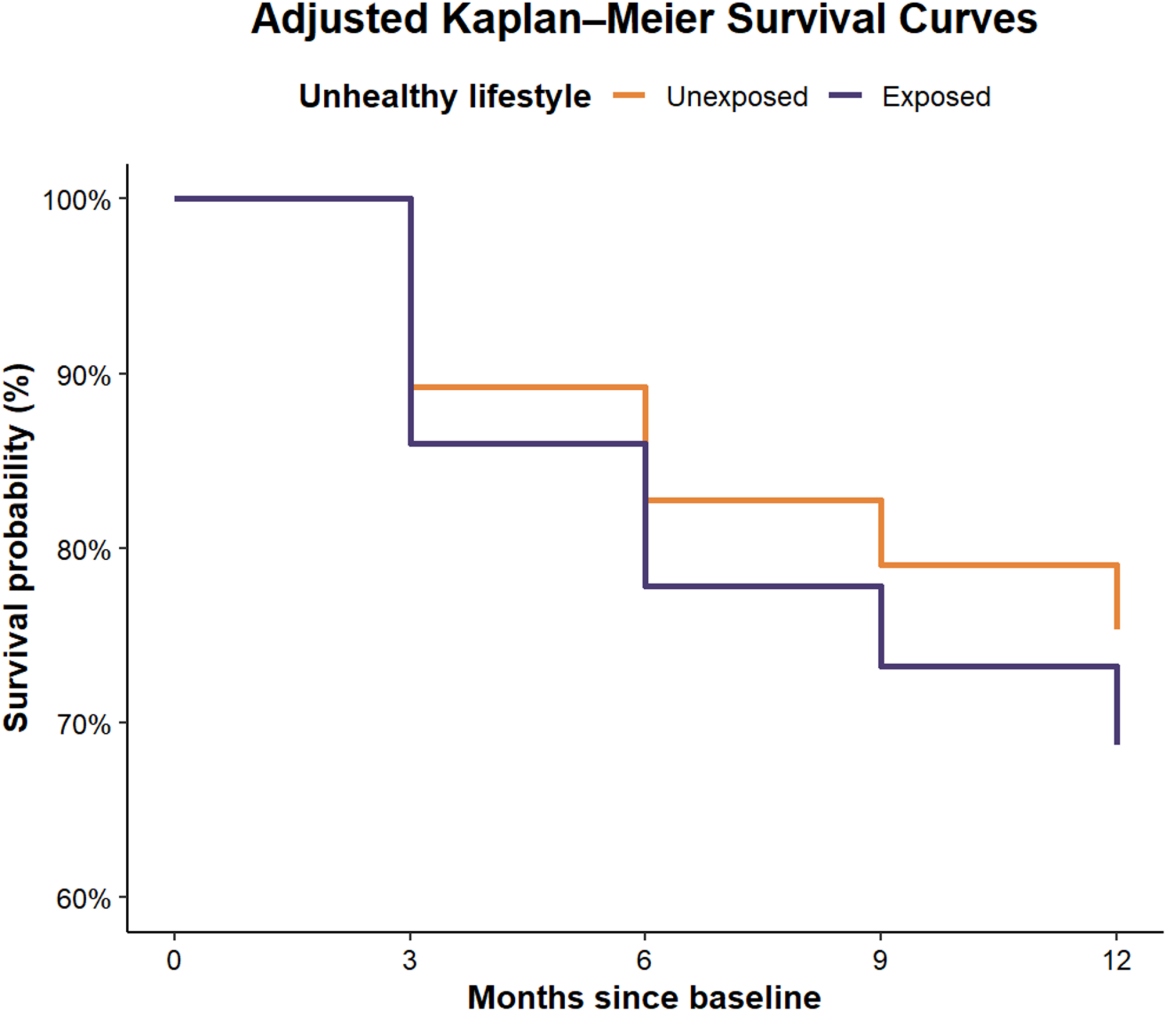
Adjusted Kaplan–Meier survival curves comparing participants exposed and unexposed to an unhealthy lifestyle at follow-up. Curves were estimated using the conditional method based on the Cox proportional hazard regression model, for the covariate levels median age (23 years), female gender, and having at least one parent with a university degree

There were 583 events among the 2918 participants unexposed to an unhealthy lifestyle (20% of the unexposed participants had ALNBP) and 135 events among the 574 participants exposed to an unhealthy lifestyle (24% of the exposed participants had ALNBP) during the one-year follow-up. Adjusted Kaplan Meier survival curves are illustrated in Fig. 2.

Crude HRR for the association between an unhealthy lifestyle and incident ALNBP was 1.32 (95% CI 1.10, 1.59). The adjusted analysis generated a HRR of 1.35 (95% CI 1.12, 1.63).

The sensitivity analysis shows that the adjusted HRR could be explained away by an unmeasured confounder associated with an unhealthy lifestyle and ALNBP by a risk ratio of 1.76 above and beyond the measured confounders, whereas weaker confounding would not do so. Likewise, the CI could be moved to include the null by an unmeasured confounder associated by a risk ratio of 1.38 above and beyond the measured confounder, whereas weaker confounding would not do so.

## Discussion

This study found an association between an unhealthy lifestyle, categorized as ≥ 3 unhealthy lifestyle behaviors (physical inactivity, high sitting time, meal skipping, risky alcohol use, risky tobacco use and risky illicit drug use), and incident ALNBP in university students, compared to < 3 unhealthy lifestyle behaviors.

The study addresses disease prevention including the assessment of health risks, in contrast to most previous research on this topic focusing on health-promotion, with the aim to encourage activities that facilitate healthy living and well-being [51]. Therefore, previous studies are not totally comparable with our study. Cohort studies by Bohman et al. (2014 and 2019) and Skillgate et al. (2017) investigated the association between a healthy lifestyle and the risk and prognosis of NBP [37–39], where a healthy lifestyle including recommended physical activity levels and sufficient fruits/vegetables intake, low alcohol consumption and non-smoking were associated with a lower risk and a better prognosis of NBP. To the best of our knowledge, only one previous cohort study has investigated the association between an unhealthy lifestyle and musculoskeletal outcomes [40]. Smedbråten et al. (2022) found that an accumulation of ≥ 4 unhealthy lifestyle behaviors (low physical activity level, sleep problems, insufficient fruit/vegetables consumption, smoking, frequent alcohol intoxication and illicit drug use) was associated with persistent musculoskeletal pain eleven years later in adolescents with musculoskeletal pain at baseline. Apart from focusing on musculoskeletal pain instead of activity-limiting musculoskeletal problems, as well as including some other lifestyle behaviors (i.e., sleep, fruits/vegetables intake and frequency of alcohol, tobacco, and drugs instead of risk use), the study by Smedbråten et al. (2022) also differs from the current study by including a higher cut-off of what is considered an unhealthy lifestyle. Given the advances in public health research of the severe and manifold consequences of having an unhealthy lifestyle [31], a cut-off of ≥ 3 instead of ≥ 4 unhealthy lifestyle behaviors was considered more appropriate. Moreover, we used a slightly different definition of an unhealthy lifestyle that was based on scientific consensus of which unhealthy lifestyle behaviors that are of highest concern for global ill-health [31], with cut-offs based on current public health recommendations [43–47].

This study includes a large sample of university students from a range of education programs, which strengthens the results’ external validity. The longitudinal study design allows investigation of a temporal, potentially causal relationship between an unhealthy lifestyle and incident ALNBP. For that purpose, students with ALNBP at baseline were excluded. Having an unhealthy lifestyle was less common among included participants (16%) compared to participants that were excluded due to having ALNBP at baseline (22%).

It is important to acknowledge that although the outcome was defined as being limited in normal daily activities due to a musculoskeletal problem, it is possible that some participants had a musculoskeletal problem that not yet had been developed to activity-limiting, but that the problem itself could impact lifestyle behaviors. For example, NBP not being so severe that it had become activity-limiting might have influenced alcohol or illicit drug use, which would have led to reverse causality with a potential overestimation of the results.

A set of covariates was included in the analysis. However, residual or unmeasured confounding of the association between an unhealthy lifestyle and ALNBP cannot be ruled out, especially since lifestyle behaviors are complex and likely related to a range of factors. An unhealthy lifestyle could be a proxy for an overall poorer health, compared to a healthy lifestyle. For example, a larger proportion of the participants exposed to an unhealthy lifestyle had moderate or severe symptoms of mental health problems as well as poor sleep quality, which are associated with both unhealthy lifestyle behaviors and NBP. It is, however, possible that mental health problems and poor sleep quality, although measured at baseline, are part of the causal pathway between an unhealthy lifestyle and ALNBP, as suggested in the DAG in Supplemental Fig. 1, in which an unhealthy lifestyle could lead to poor sleep or mental health problems (or vice versa). Including these factors in the statistical model could, potentially and erroneously, adjust away the association, which was the rationale for not including factors other than demographics, preceding both the exposure and outcome, in the final model. The E-value sensitivity analysis indicates that unmeasured or residual confounders would need to increase the risk of an unhealthy lifestyle and ALNBP by 76% to move the HRR point estimate to the null. Residual confounding could, hence, potentially explain away the observed association, but needs to be moderately associated with an unhealthy lifestyle and ALNBP, even after adjustment of potential confounders. Given the complexity of lifestyle behaviors, it is not impossible that other factors (e.g. genetics) could confound the association, hence the result would be explained by confounding.

There is a risk of misclassification of the separate lifestyle behaviors used to categorize the exposure, where the association between an unhealthy lifestyle and incident ALNBP could have been under or overestimated. The reason for the categorization of exposed as ≥ 3 unhealthy lifestyle behaviors was to capture the lifestyle as an overall behavior and not to assess single lifestyle behaviors, and not to have too few exposed participants in the statistical analysis. Since self-reported lifestyle behaviors tend to be reported in accordance with social desirability, objective methods (e.g. physical activity assessed with an accelerometer) would likely have given more accurate measures. There is, furthermore, a risk of loss of in-depth information by dichotomization of the variables included in the unhealthy lifestyle exposure, such as physical activity where a potential U-shaped association with musculoskeletal problems [52] was not considered. The instruments used to measure physical activity and risky use of alcohol, tobacco and illicit drug use have shown acceptable, yet not excellent, validity and reliability. Also, it is possible that a healthy lifestyle was not captured with the separate lifestyle behaviors included. For example, it would have been more informative to measure adherence to the Nordic Nutrition Recommendations (i.e. intake frequency of fruits/vegetables, whole-grain, candy, sugar-sweetened beverages etc.) than meal frequency for capturing unhealthy dietary habits. Also, it is possible that participants changed their lifestyle behaviors during the one-year follow-up, which could lead to a dilution of the found associations. Finally, the chosen cut-off for an unhealthy lifestyle might not be optimal, since having also < 3 unhealthy lifestyle behaviors could be considered an unhealthy lifestyle from a medical perspective (e.g. a risky alcohol use and smoking one package of cigarettes per day) even if having also other healthy lifestyle behaviors. From this perspective, the associations might be underestimated. Any misclassification of the exposure would, however, likely be non-differential and dilute a true association.

This study had an acceptable three-month follow-up rate of on average 72%, yet potential selection bias might threaten the study’s validity, given that it is unknown whether the non-responders would have different rate of incident ALNBP. Among those lost to follow-up at the last time-point of measurements, 20% were exposed to an unhealthy lifestyle at baseline compared to 14% among those successfully followed. The exposure status is similar but may have introduced selection bias if the loss to follow-up are related to the outcome, i.e. if the non-responders also had a higher rate of incident ALNBP, the results may be somewhat biased.

Given that unhealthy lifestyle behaviors tend to cluster [33–36], our aim was to investigate an unhealthy lifestyle as an overall behavioral pattern. The mechanisms behind the association of an unhealthy lifestyle and ALNBP are likely multi-factorial, where biological, social, and psychological mechanisms interact, and should suggestively be further investigated. The findings of this study add to the existing knowledge about the role of lifestyle behaviors for common musculoskeletal problems with high societal relevance, and advocate for future studies investigating the role of lifestyle in prevention of ALNBP in university students. In the ongoing work to better understand the complex etiology of ALNBP, the findings from this study may offer valuable insights that support the future health of university students. Given the well-established benefits of a healthy lifestyle across various health domains, continued efforts to promote healthy living remain essential.

## Conclusion

An unhealthy lifestyle, categorized as ≥ 3 unhealthy lifestyle behaviors, is associated with incident activity-limiting neck/back problems in university students.

## Supplementary Information

Supplementary Material 1.

## Data Availability

The datasets used and analyzed are not publicly available due to collection of sensitive personal information but are available on reasonable request by contacting the study guarantor Eva Skillgate at eva.skillgate@shh.se.

## Abbreviations

ALNBP: Activity-limiting neck/back problems
ASSIST: Alcohol, Smoking and Substance Involvement Screening Test
CI: Confidence interval
DAG: Directed acyclic graph
HRR: Hazard rate ratio
NBP: Neck and back pain
NMQ: Nordic Musculoskeletal Questionnaire
SD: Standard deviation
SUN-study: Sustainable UNiversity Life study

## References

[1] oGlobal burden of 369 diseases and injuries in 204 countries and territories, 1990-2019: a systematic analysis for the Global Burden of Disease Study 2019. Lancet. 2020;396(10258):1204-22. 33069326 10.1016/S0140-6736(20)30925-9 PMC7567026

[2] Global, regional, and national burden of low back pain, 1990-2020, its attributable risk factors, and projections to 2050: a systematic analysis of the Global Burden of Disease Study 2021. Lancet Rheumatol. 2023;5(6):e316-e29. 10.1016/S2665-9913(23)00098-X PMC10234592 37273833

[3] Global, regional, and national burden of neck pain, 1990-2020, and projections to 2050: a systematic analysis of the Global Burden of Disease Study 2021. Lancet Rheumatol. 2024;6(3):e142-e55. 38383088 10.1016/S2665-9913(23)00321-1 PMC10897950

[4] Cieza A, Causey K, Kamenov K, Hanson SW, Chatterji S, Vos T. Global estimates of the need for rehabilitation based on the Global Burden of Disease study 2019: a systematic analysis for the Global Burden of Disease Study 2019. Lancet. 2021;396(10267):2006-17. 33275908 10.1016/S0140-6736(20)32340-0 PMC7811204

[5] Mokdad AH, Forouzanfar MH, Daoud F, Mokdad AA, El Bcheraoui C, Moradi-Lakeh M, et al. Global burden of diseases, injuries, and risk factors for young people's health during 1990-2013: a systematic analysis for the Global Burden of Disease Study 2013. Lancet. 2016;387(10036):2383-401. 27174305 10.1016/S0140-6736(16)00648-6

[6] Grasdalsmoen M, Engdahl B, Fjeld MK, Steingrímsdóttir ÓA, Nielsen CS, Eriksen HR, et al. Physical exercise and chronic pain in university students. PLoS ONE. 2020;15(6):e0235419–e. 32589694 10.1371/journal.pone.0235419 PMC7319292

[7] Campbell A, Wang D, Martin K, Côté P. The one-week prevalence of neck pain and low back pain in post-secondary students at two Canadian institutions. Chiropr Man Therap. 2023;31(1):23. 37525206 10.1186/s12998-023-00496-y PMC10391772

[8] Oksanen AM, Laimi K, Löyttyniemi E, Kunttu K. Trends of weekly musculoskeletal pain from 2000 to 2012: national study of Finnish university students. Eur J Pain. 2014;18(9):1316–22. 24687865 10.1002/j.1532-2149.2014.492.x

[9] Gilkey DP, Keefe TJ, Peel JL, Kassab OM, Kennedy CA. Risk factors associated with back pain: a cross-sectional study of 963 college students. J Manipulative Physiol Ther. 2010;33(2):88–95. 20170773 10.1016/j.jmpt.2009.12.005

[10] Amelot A, Mathon B, Haddad R, Renault M-C, Duguet A, Steichen O. Low back pain among medical students: a burden and an impact to consider! Spine. 2019;44(19):1. 31261281 10.1097/BRS.0000000000003067

[11] Serbic D, Friedrich C, Murray R. Psychological, social and academic functioning in university students with chronic pain: a systematic review. J Am Coll Health. 2023;71(9):2894–908. 34871522 10.1080/07448481.2021.2006199

[12] Maayah MF, Nawasreh ZH, Gaowgzeh RAM, Neamatallah Z, Alfawaz SS, Alabasi UM. Neck pain associated with smartphone usage among university students. PLoS ONE. 2023;18(6):e0285451. 37352232 10.1371/journal.pone.0285451 PMC10289365

[13] Parto DN, Wong AY, Macedo L. Prevalence of musculoskeletal disorders and associated risk factors in Canadian university students. BMC Musculoskelet Disord. 2023;24(1):501. 37337246 10.1186/s12891-023-06630-4 PMC10278339

[14] Alshehri MM, Alqhtani AM, Gharawi SH, Sharahily RA, Fathi WA, Alnamy SG, et al. Prevalence of lower back pain and its associations with lifestyle behaviors among college students in Saudi Arabia. BMC Musculoskelet Disord. 2023;24(1):646. 37568153 10.1186/s12891-023-06683-5 PMC10416365

[15] Alghamdi MS, Alghamdi AF, Almalawi AM, Alsulami RA, Hazazi HA, Al Ghashmari AA, et al. The association between neck pain and psychological distress experienced by King Abdulaziz university students: A Cross-Sectional study. Cureus. 2023;15(3):e35685. 37012948 10.7759/cureus.35685 PMC10066660

[16] Al-Hadidi F, Bsisu I, AlRyalat SA, Al-Zu’bi B, Bsisu R, Hamdan M, et al. Association between mobile phone use and neck pain in university students: a cross-sectional study using numeric rating scale for evaluation of neck pain. PLoS ONE. 2019;14(5):e0217231. 31107910 10.1371/journal.pone.0217231 PMC6527223

[17] Daniyal M, Javaid SF, Hassan A, Khan MAB. The relationship between cellphone usage on the physical and mental wellbeing of university students: a cross-sectional study. Int J Environ Res Public Health. 2022;19(15):9352. 35954709 10.3390/ijerph19159352 PMC9368281

[18] Elsiddig AI, Altalhi IA, Althobaiti ME, Alwethainani MT, Alzahrani AM. Prevalence of neck and shoulder pain among Saudi universities’ students who are using smartphones and computers. J Family Med Prim Care. 2022;11(1):194–200. 35309622 10.4103/jfmpc.jfmpc_1138_21 PMC8930126

[19] Hanphitakphong P, Keeratisiroj O, Thawinchai N. Smartphone addiction and its association with upper body musculoskeletal symptoms among university students classified by age and gender. J Phys Ther Sci. 2021;33(5):394–400. 34083877 10.1589/jpts.33.394 PMC8165358

[20] Walankar PP, Kemkar M, Govekar A, Dhanwada A. Musculoskeletal pain and risk factors associated with smartphone use in university students. Indian J Occup Environ Med. 2021;25(4):220–4. 35197674 10.4103/ijoem.ijoem_351_20 PMC8815661

[21] Fernández-Villa T, Alguacil Ojeda J, Almaraz Gómez A, Cancela Carral JM, Delgado-Rodríguez M, García-Martín M, et al. Problematic internet use in university students: associated factors and differences of gender. Adicciones. 2015;27(4):265–75. 26706809

[22] Alsaadi SM. Musculoskeletal pain in undergraduate students is significantly associated with psychological distress and poor sleep quality. Int J Environ Res Public Health. 2022;19:21. 10.3390/ijerph192113929 PMC9658124 36360807

[23] Hamaoka K, Ashizawa R, Hida M, Suganuma I, Yoshimoto Y. Chronic lumbar pain and insomnia in College-Aged students. Healthcare. 2022. 10.3390/healthcare10040701 35455878 10.3390/healthcare10040701 PMC9031783

[24] Johansson F, Billquist J, Andreasson H, Jensen I, Onell C, Berman AH, et al. Study environment and the incidence of mental health problems and activity-limiting musculoskeletal problems among university students: the SUN cohort study. BMJ Open. 2023;13(9):e072178. 37709330 10.1136/bmjopen-2023-072178 PMC10503358

[25] Kanchanomai S, Janwantanakul P, Pensri P, Jiamjarasrangsi W. A prospective study of incidence and risk factors for the onset and persistence of low back pain in Thai university students. Asia Pac J Public Health. 2015;27(2):Np106–15. 22186386 10.1177/1010539511427579

[26] Mazaheri-Tehrani S, Arefian M, Abhari AP, Riahi R, Vahdatpour B, Baradaran Mahdavi S, et al. Sedentary behavior and neck pain in adults: a systematic review and meta-analysis. Prev Med. 2023;175:107711. 37775083 10.1016/j.ypmed.2023.107711

[27] Castro O, Bennie J, Vergeer I, Bosselut G, Biddle SJH. How sedentary are university students? A systematic review and meta-analysis. Prev Sci. 2020;21(3):332–43. 31975312 10.1007/s11121-020-01093-8

[28] Deforche B, Van Dyck D, Deliens T, De Bourdeaudhuij I. Changes in weight, physical activity, sedentary behaviour and dietary intake during the transition to higher education: a prospective study. Int J Behav Nutr Phys Act. 2015;12:16. 25881147 10.1186/s12966-015-0173-9 PMC4332914

[29] Davoren MP, Demant J, Shiely F, Perry IJ. Alcohol consumption among university students in Ireland and the united Kigdom from 2002 to 2014: a systematic review. BMC Public Health. 2016;16:173. 26895824 10.1186/s12889-016-2843-1 PMC4759952

[30] Schmidt M. Predictors of self-rated health and lifestyle behaviours in Swedish university students. Glob J Health Sci. 2012;4(4):1–14. 22980336 10.5539/gjhs.v4n4p1 PMC4776941

[31] World Health Organization. Noncommunicable diseases. Geneva: World Health Organization. 2023. Available from: https://www.who.int/news-room/fact-sheets/detail/noncommunicable-diseases. Cited 2024 May 10.

[32] Socialstyrelsen. Nationella riktlinjer för prevention och behandling vid ohälsosamma levnadsvanor: Stöd för styrning och ledning. Stockholm: Socialstyrelsen. 2018. Available from: https://www.socialstyrelsen.se/globalassets/sharepoint-dokument/artikelkatalog/nationella-riktlinjer/2018-6-24.pdf. Cited 2024 May 10.

[33] Meader N, King K, Moe-Byrne T, Wright K, Graham H, Petticrew M, et al. A systematic review on the clustering and co-occurrence of multiple risk behaviours. BMC Public Health. 2016;16:657. 27473458 10.1186/s12889-016-3373-6 PMC4966774

[34] Bennasar-Veny M, Yañez AM, Pericas J, Ballester L, Fernandez-Dominguez JC, Tauler P, et al. Cluster analysis of health-related lifestyles in university students. Int J Environ Res Public Health. 2020. 10.3390/ijerph17051776. 32182922 10.3390/ijerph17051776 PMC7084566

[35] Noble N, Paul C, Turon H, Oldmeadow C. Which modifiable health risk behaviours are related? A systematic review of the clustering of Smoking, Nutrition, alcohol and physical activity (‘SNAP’) health risk factors. Prev Med. 2015;81:16–41. 26190368 10.1016/j.ypmed.2015.07.003

[36] Murphy JJ, MacDonncha C, Murphy MH, Murphy N, Timperio A, Leech RM, et al. Identification of health-related behavioural clusters and their association with demographic characteristics in Irish university students. BMC Public Health. 2019;19(1):121. 30691428 10.1186/s12889-019-6453-6 PMC6350296

[37] Bohman T, Holm LW, Hallqvist J, Pico-Espinosa OJ, Skillgate E. Healthy lifestyle behaviour and risk of long-duration troublesome neck pain among men and women with occasional neck pain: results from the Stockholm public health cohort. BMJ Open. 2019;9(11):e031078. 31748298 10.1136/bmjopen-2019-031078 PMC6887003

[38] Skillgate E, Pico-Espinosa OJ, Hallqvist J, Bohman T, Holm LW. Healthy lifestyle behavior and risk of long duration troublesome neck pain or low back pain among men and women: results from the Stockholm public health cohort. Clin Epidemiol. 2017;9:491–500. 29066933 10.2147/CLEP.S145264 PMC5644563

[39] Bohman T, Alfredsson L, Jensen I, Hallqvist J, Vingård E, Skillgate E. Does a healthy lifestyle behaviour influence the prognosis of low back pain among men and women in a general population? A population-based cohort study. BMJ Open. 2014;4(12):e005713. 25550292 10.1136/bmjopen-2014-005713 PMC4281558

[40] Smedbråten K, Grotle M, Jahre H, Richardsen KR, Småstuen MC, Skillgate E, et al. Lifestyle behaviour in adolescence and musculoskeletal pain 11 years later: the Trøndelag health study. Eur J Pain. 2022;26(9):1910–22. 35851511 10.1002/ejp.2012 PMC9545098

[41] Pronk NP, Lowry M, Kottke TE, Austin E, Gallagher J, Katz A. The association between optimal lifestyle adherence and short-term incidence of chronic conditions among employees. Popul Health Manag. 2010;13(6):289–95. 21090987 10.1089/pop.2009.0075

[42] Edlund K, Sundberg T, Johansson F, Onell C, Rudman A, Holm LW, et al. Sustainable university life (SUN) study: protocol for a prospective cohort study of modifiable risk and prognostic factors for mental health problems and musculoskeletal pain among university students. BMJ Open. 2022;12(4):e056489. 35379630 10.1136/bmjopen-2021-056489 PMC8980731

[43] Bull FC, Al-Ansari SS, Biddle S, Borodulin K, Buman MP, Cardon G, et al. World health organization 2020 guidelines on physical activity and sedentary behaviour. Br J Sports Med. 2020;54(24):1451–62. 33239350 10.1136/bjsports-2020-102955 PMC7719906

[44] Larsson K, Kallings LV, Ekblom Ö, Blom V, Andersson E, Ekblom MM. Criterion validity and test-retest reliability of SED-GIH, a single item question for assessment of daily sitting time. BMC Public Health. 2019;19(1):17. 30611226 10.1186/s12889-018-6329-1 PMC6321678

[45] Pendergast FJ, Livingstone KM, Worsley A, McNaughton SA. Correlates of meal skipping in young adults: a systematic review. Int J Behav Nutr Phys Act. 2016;13(1):125. 27905981 10.1186/s12966-016-0451-1 PMC5133750

[46] Humeniuk R-E, AR, Poznyak V, Monteiro M. The Alcohol, smoking and substance involvement screening test (ASSIST): manual for use in primary care. Geneva: World Health Organization; 2010.

[47] Humeniuk R, Ali R, Babor TF, Farrell M, Formigoni ML, Jittiwutikarn J, et al. Validation of the alcohol, smoking and substance involvement screening test (ASSIST). Addiction. 2008;103(6):1039–47. 18373724 10.1111/j.1360-0443.2007.02114.x

[48] Kuorinka I, Jonsson B, Kilbom A, Vinterberg H, Biering-Sorensen F, Andersson G, et al. Standardised nordic questionnaires for the analysis of musculoskeletal symptoms. Appl Ergon. 1987;18(3):233–7. 15676628 10.1016/0003-6870(87)90010-x

[49] VanderWeele TJ, Ding P. Sensitivity analysis in observational research: introducing the E-value. Ann Intern Med. 2017;167(4):268–74. 28693043 10.7326/M16-2607

[50] Henry JD, Crawford JR. The short-form version of the depression anxiety stress scales (DASS-21): construct validity and normative data in a large non-clinical sample. Br J Clin Psychol. 2005;44(Pt 2):227–39. 16004657 10.1348/014466505X29657

[51] Caron RM, Noel K, Reed RN, Sibel J, Smith HJ. Health promotion, health protection, and disease prevention: challenges and opportunities in a dynamic landscape. Am J Prev Med. 2024;3(1):100167. 10.1016/j.focus.2023.100167 PMC10749873 38149078

[52] Heneweer H, Vanhees L, Picavet HS. Physical activity and low back pain: a U-shaped relation? Pain. 2009;143(1–2):21–5. 19217208 10.1016/j.pain.2008.12.033

