# Supplementary material for: An unhealthy lifestyle and incident activity-limiting neck and back problems in university students: the Sustainable UNiversity Life (SUN) study
Source: BMC Public Health. 2025 Nov 6;25:3820. doi: 10.1186/s12889-025-25478-y (PMC12590823; doi:10.1186/s12889-025-25478-y)
Supplement: Supplementary file 1 — Supplementary Material 1. [file 12889_2025_25478_MOESM1_ESM.docx]

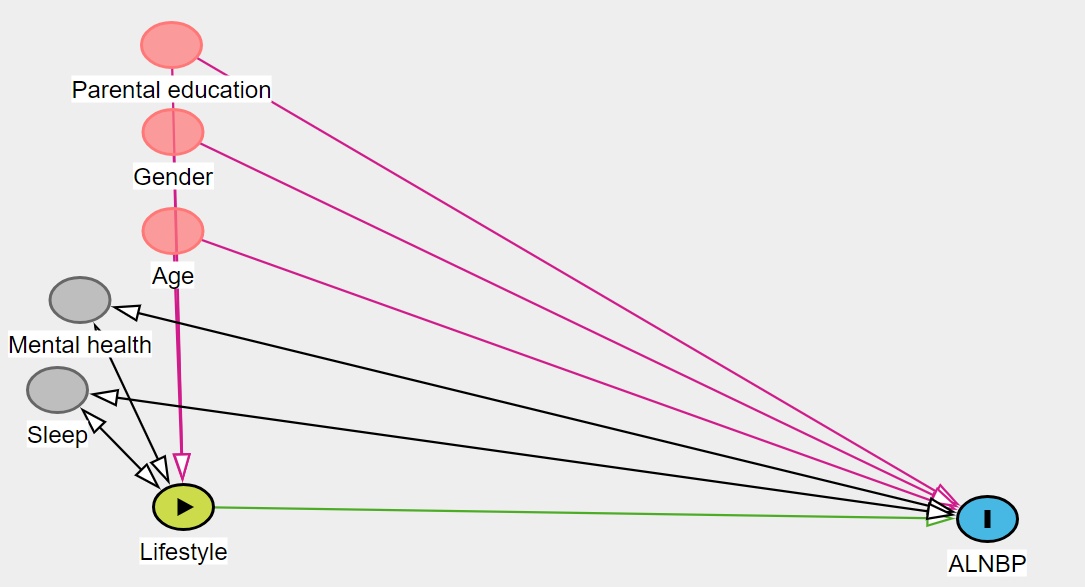


**Supplemental figure 1.** Directed acyclic graph of the association between lifestyle and activity-limiting neck/back problems (ALNBP). Pink circles indicate variables suggested to be adjusted for as confounders, whereas grey circles are suggested not to be adjusted for since these may be part of the causal pathway.
